# Clozapine cessation

**DOI:** 10.1192/j.eurpsy.2022.1884

**Published:** 2022-09-01

**Authors:** H. Ktari, A. Ouertani, S. Madouri, A. Aissa, Y. Zgueb, U. Ouali, R. Jomli

**Affiliations:** 1 Razi hospital, Psychiatry A Department, Manouba, Tunisia; 2 Razi Hospital, Psychiatry A, Manouba, Tunisia; 3 Razi hospital, Psychiatry A Department, manouba, Tunisia

## Abstract

**Introduction:**

Approximately 30% of individuals diagnosed with schizophrenia suffer from treatment-resistant or refractory schizophrenia. The gold standard for treatment is clozapine. However, a significant number of patients discontinue clozapine treatment and this carries a poor prognosis.

**Objectives:**

This study explores patients’ motives for cessation of clozapine therapy and its prevalence.

**Methods:**

A longitudinal, retrospective and descriptive study on a period of 20 years, at the psychiatry department A of the Razi hospital in Tunisia. Data was collected from the medical files of patients trated by clozapine using a pre-established sheet.

**Results:**

The studied sample included 64 patient records. Treatment with clozapine was stopped spontaneously or following a medical decision in 37 patients (57.8%). The total number of clozapine stops in these 37 patients was 70. Indeed, each one of these patients had stopped treatment at least once. Clozapine was discontinued by some patients in the study sample for poor compliance(45.9%), for adverse side effects of treatment (16.2%) and by treating physicians for poor response treatment (8.1%). Clozapine was discontinued by 11 patients for hematological adverse reactions, representing 27.9% of the total number of clozapine discontinuations. Withdrawal of clozapine was indicated in 2 cases of agranulocytosis(18.2%), in 2 cases of moderate neutropenia(18.2%), in 3 cases of eosinophilia (27.2%), in 3 cases of thrombocytopenia (27.2%) and in 1 case of severe anemia (9.2%).

**Conclusions:**

Clozapine discontinuation was essentially caused by poor patients’ observation and hematological adverse reactions appearance.Future research should seek to further investigate clozapine cessation factors in order to better benefit from the medical virtues of this molecule.

**Disclosure:**

No significant relationships.

